# Differential Antioxidant Response to Supplemental UV-B Irradiation and Sunlight in Three Basil Varieties

**DOI:** 10.3390/ijms242015350

**Published:** 2023-10-19

**Authors:** Sonja Milić Komić, Bojana Živanović, Jelena Dumanović, Predrag Kolarž, Ana Sedlarević Zorić, Filis Morina, Marija Vidović, Sonja Veljović Jovanović

**Affiliations:** 1 Institute for Multidisciplinary Research, Department of Life Science, University of Belgrade, Kneza Višeslava 1, 11030 Belgrade, Serbia; sonjamilic@imsi.bg.ac.rs (S.M.K.); bojana.zivanovic@imsi.bg.ac.rs (B.Ž.); ana.sedlarevic@imsi.bg.ac.rs (A.S.Z.); 2 Department of Analytical Chemistry, Faculty of Chemistry, University of Belgrade, 11158 Belgrade, Serbia; dumanovicjelena@gmail.com; 3 Institute of Physics Belgrade, University of Belgrade, 11080 Belgrade, Serbia; kolarz@ipb.ac.rs; 4 Biology Center of the Czech Academy of Sciences, Institute of Plant Molecular Biology, Department of Plant Biophysics and Biochemistry, Branišovska 31/1160, 370 05 Ceske Budejovice, Czech Republic; morina@umbr.cas.cz; 5 Institute of Molecular Genetics and Genetic Engineering, Laboratory for Plant Molecular Biology, University of Belgrade, Vojvode Stepe 444a, 11042 Belgrade, Serbia; mvidovic@imgge.bg.ac.rs

**Keywords:** ascorbate, epidermal flavonoids, hydrogen peroxide, *Ocimum basilicum* var. Genovese, *Ocimum* × *citriodorum*, *Ocimum basilicum* var. *purpurascens*, polyphenols, supplemented and ecologically relevant UV-B irradiation, total leaf antioxidant capacity

## Abstract

Three basil plant varieties (*Ocimum basilicum* var. Genovese, *Ocimum* × *citriodorum*, and *Ocimum basilicum* var. *purpurascens*) were grown under moderate light (about 300 µmol photons m^−2^ s^−1^) in a glasshouse or growth chamber and then either transferred to an open field (average daily dose: 29.2 kJ m^−2^ d^−1^) or additionally exposed to UV-B irradiation in a growth chamber (29.16 kJ m^−2^ d^−1^), to reveal the variety-specific and light-specific acclimation responses. Total antioxidant capacity (TAC), phenolic profile, ascorbate content, and class III peroxidase (POD) activity were used to determine the antioxidant status of leaves under all four light regimes. Exposure to high solar irradiation at the open field resulted in an increase in TAC, total hydroxycinnamic acids (HCAs, especially caffeic acid), flavonoids, and epidermal UV-absorbing substances in all three varieties, as well as a two-fold increase in the leaf dry/fresh weight ratio. The supplemental UV-B irradiation induced preferential accumulation of HCAs (rosmarinic acid) over flavonoids, increased TAC and POD activity, but decreased the ascorbate content in the leaves, and inhibited the accumulation of epidermal flavonoids in all basil varieties. Furthermore, characteristic leaf curling and UV-B-induced inhibition of plant growth were observed in all basil varieties, while a pro-oxidant effect of UV-B was indicated with H_2_O_2_ accumulation in the leaves and spotty leaf browning. The extent of these morphological changes, and oxidative damage depended on the basil cultivar, implies a genotype-specific tolerance mechanism to high doses of UV-B irradiation.

## 1. Introduction

Plants are inevitably exposed to seasonal and diurnal variations in light intensity and quality as a result of variations in the ratio of UV-A/UV-B/photosynthetically active radiation (PAR, 400–700 nm) [1,2]. Ultraviolet (UV) irradiation accounts for only a small fraction of total solar radiation, about 6% UV-A (315–400 nm) and 0.5% UV-B (290–315 nm); nevertheless, it can induce a variety of responses in plants. Since the 1980s, the depletion of the stratospheric ozone layer has led to an increase of up to 14% in UV-B radiation reaching the Earth’s surface [3]. The Montreal Protocol and its amendments were remarkably effective in saving the Earth’s stratospheric ozone layer and led to its restoration. Nevertheless, there is a strong link between ozone depletion and climate changes caused by increasing greenhouse gases. Recent studies predict a 3–8% increase in the UV index over the tropics or mid-latitudes by 2100, depending on the greenhouse gas scenario, cloud cover, and aerosol concentration used for modelling [4,5,6]. The predicted decrease in mean cloud cover over the Mediterranean region, due to climate change, could also lead to an increase in the intensity of UV radiation over the Mediterranean region in the near future [7]. The meteorological data for this study were collected in Belgrade (Serbia), situated at the 45th parallel of northern latitude humid subtropical and humid continental climate, exhibiting four distinct seasons and precipitation evenly distributed throughout the year. Malinović-Milićević and co-authors showed that the average daily UV-B dose ranged from 2.062 kJ m^−2^ (December) to 58.773 kJ m^−2^ (June) and estimated a 3.7% increase in UV-B radiation intensity per decade in the territory of Vojvodina (northern region of Serbia) [8]. Similar UV-B doses have been measured in Cyprus, where radiation doses vary from 70 kJ m^−2^ in Larnaca to 55 kJ m^−2^ in Athalassa, depending on the climate regime of the island [9]. High UV-B radiation can cause DNA damage and/or induce the generation of reactive oxygen species (ROS), which can oxidize proteins and membranes, and inhibit photosynthesis and growth [10]. However, plants rarely show signs of damage even though they are constantly exposed to natural UV-B irradiation [11]. There are numerous studies with conflicting results on the effect of UV-B irradiation on plants, which can be explained with different ratios of UV-B/UV-A/PAR, and unnaturally high UV-B doses applied in the experiments, as well as species/genotype-specific sensitivity to UV-B radiation, different experimental conditions, and previous acclimation periods [12]. Moreover, high PAR, combined with other limiting environmental factors such as water and/or nutrient deficiencies, can overcome the capacity of photosynthetic assimilation and energy dissipation processes and provoke the generation of ROS, resulting in photo-oxidative damage to the photosynthetic apparatus [13,14].

Plants respond to light by developing numerous photoprotective mechanisms against the deleterious effects of excess PAR and UV-B, of which the antioxidant network and redox signaling pathways play an important role in maintaining cellular redox homeostasis [15,16]. The most efficient and rapid photoprotective process of the photosynthetic apparatus is the dissipation of excess excitation energy in photosystem II (PSII) with heat, which is controlled with the trans-thylakoid proton gradient and zeaxanthin formation [17,18]. As part of the acclimation mechanisms to PAR and UV radiation, the enhanced biosynthesis of phenolics can also be considered a photosynthetic energy escape valve, with 20% of the fixed carbon in photosynthesis being directed into the phenylpropanoid pathway [19]. The accumulation of phenolics, particularly flavonoids, is considered a hallmark of the UV-B response in plants [2]. Polyphenols are the largest and most diverse group of secondary metabolites that play a crucial role in the dynamic interactions between plants and the environment. Under full sunlight, plants tend to accumulate UV-absorbing phenolics, especially flavonols, in the epidermal layer of exposed leaves, providing them with an effective protective shield against potentially harmful UV radiation [20]. In addition to their protective role in attenuating UV radiation and PAR, flavonoids and anthocyanidins are potent antioxidants, whose efficiency in ROS scavenging is mainly determined with their structure [21]. Flavonoids and anthocyanidins with *ortho*-dihydroxyl substitution in the B ring have almost four times higher antioxidant activity than the other polyphenols. Anthocyanins exhibit multiple functional roles in plants that have to cope with a variety of abiotic and biotic stress factors [22,23]. In addition to their absorption in the blue and green regions of the spectrum, they are extremely effective antioxidants that can scavenge ROS in vitro and act as substrates for PODs and participate in H_2_O_2_ scavenging [24]. Anthocyanins are commonly induced with high light intensity, low nitrogen content, and low temperatures. On the other hand, both UV-B and high PAR intensities induce phenylpropanoid accumulation in the epidermal layers, such as quercetin and catechin, which also exhibit a strong antioxidant potential through direct interaction with ROS [25].

In contrast to the deleterious effects of high UV-B irradiation, low UV-B levels trigger a UV-B-specific signaling pathway that mediates photomorphogenic responses and the development of the so-called “UV-acclimated phenotype” with shorter petioles and shorter, narrower, and/or thicker leaf blades, as well as leaf curling [26]. The photomorphogenic UV-B signaling pathway is mediated with the UV-B-specific component UV RESISTANCE LOCUS8 (UVR8). Both UVR8 and CONSTITUTIVE PHOTOMORPHOGENESIS 1 (COP1) are required for UV-B-induced expression of the ELONGATED HYPOCOTYL5 (HY5) transcription factor, which plays a central role in regulating genes involved in photomorphogenic UV-B responses [2].

Basil (*Ocimum basilicum* L.) is a widely used aromatic herbaceous plant in nutrition, as well as in traditional medicine, pharmaceuticals, perfumes, and cosmetics [27,28]. Traditionally, basil was grown in an open field. However, in response to increasing market demand, and unpredictable weather conditions, basil cultivation has strongly shifted to controlled environment agriculture, such as glasshouses, high tunnels, and vertical indoor farms for herb production [29,30]. Light intensity and quality have a significant impact on the basil growth rate, morphology, and anatomical characteristics [31,32,33]. Considering the increased demand for high-quality basil (particularly fresh leaf material), special attention is paid to the cultivation treatment, especially the beneficial effects of low UV radiation on the functional properties, antioxidant value, and content of secondary metabolites [34,35]. In different basil varieties (purple and green), competition in the induction of either flavonoid or anthocyanin metabolic pathways depending on light quality and intensity has been shown [36]. In addition, purple basil varieties have attracted increasing interest in the last decade due to their much higher content of health-promoting substances compared to green varieties, especially when the plants are grown under limited light intensity [37]. A comparison of green and red basil leaves developed at full sunlight showed higher photosynthetic performance and higher stomatal conductance in red leaves than in green ones at a given time point [36]. However, to our knowledge, dynamics of the accumulation of UV screening compounds in basil have not been investigated. Moreover, varieties of *O. basilicum* with constitutively different contents of anthocyanins and phenolics represent a suitable system to investigate the acclimation response to different UV/PAR ratios. Comparative analyses of green vs. purple leaf response of the same age within the same species to light treatments can reveal the functional role of anthocyanins, which is still debatable as discussed by Landi and co-authors [38]. In addition, lemon basil differs from other varieties not only by its characteristic aroma and high content of biologically active polyphenolic compounds, but also by its particular morphology, characterized by a small stature, early flowering, and narrow leaves [39].

In this study, we aimed to distinguish between UV-B and high PAR effects on their pro-oxidant effects as well as on ascorbate and polyphenol contents, total and epidermal, in the leaves of three commonly consumed basil varieties: “Genovese” (*Ocimum basilicum* Genovese, GB), “Lemon” (*Ocimum* × *citriodorum*, CB), and “Purple” (*Ocimum basilicum* purpurascens, PB). We compared two green-leaf varieties and one purple-leaf cultivar, to investigate the functional role of anthocyanins in photoprotection (including excessive PAR and UV-B radiation). Based on the physicochemical properties of anthocyanins and other abundant phenylpropanoids in the leaves of three basil varieties, we hypothesized that the purple-leaf cultivar would be more tolerant to high solar radiation, and show fewer symptoms of oxidative stress damage. Furthermore, we aimed to find out whether the varieties differ in their dynamics of acclimation response to different UV/PAR ratios (supplemental UV-B irradiation, and full sun irradiance). To achieve this, the analyses of phenolic profiles, dynamics of accumulation of epidermal UV-absorbing compounds, and redox status were performed.

## 2. Results

### 2.1. Effects of Light Regimes on Growth

After 1 month of growth in the glasshouse (GH) with complete attenuation of UV irradiation and 84% of solar PAR, half of the basil plants were transferred to an open field (OF). Upon transferring the basil plants from the glasshouse to full sun for 15 days in the open field (the average daily UV-B dose was 29.20 kJ m^−2^), all three varieties showed differences in the fresh/dry weight ratio (FW/DW) compared to glasshouse plants (experiment I; Table 1). The FW/DW ratios were lower in plants grown in the open field compared to the glasshouse plants regardless of variety, with the most pronounced difference in *Ocimum basilicum* var. *purpurascens* (PB). However, only “lemon” basil (CB), DW, and, to a lesser extent, FW increased in the open field compared to the glasshouse, while growth inhibition (evidenced in FW) was only observed in “Genovese” basil (GB) (Table 1). In addition, 1 week after full sun exposure, leaf thickening was observed in “lemon” basil (CB).

**Figure 1:**
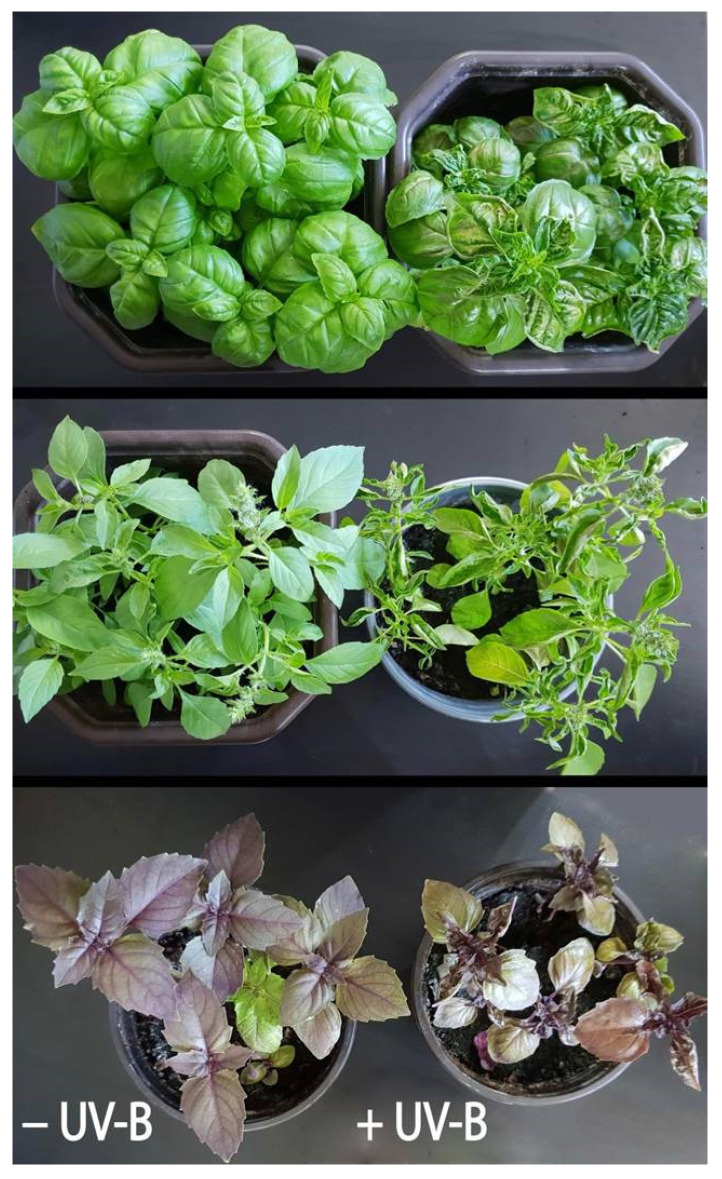
Representative images of three basil varieties (five plants for each variety) grown at constant PAR and artificial UV-B radiation (1.35 W m^−2^) 6 h per day after 8 days of treatment. *Ocimum basilicum* var. Genovese (GB) (**top**), *Ocimum* × *citriodorum* (CB) (**middle**), and *Ocimum basilicum* var. *purpurascens* (PB) (**bottom**).

In experiment II, 1-month-old plants were grown in a growth chamber (GC) under controlled conditions (PAR, 300 ± 50 μmol m^−2^ s^−1^), and half of the plants of all three varieties were exposed to supplemental UV-B irradiation (1.35 W m^−2^, i.e., 29.16 kJ m^−2^) for 6 h per day (UV-B). After only 3 days, the first small brown blotches/spots, characteristic of UV-induced damage, were noticed on the directly exposed leaves. Over the next 5 days, the damaged leaf areas increased in size and became necrotic and the young leaves began to curl, especially in the Genovese variety, which appeared to be the most sensitive variety (Figure 1). Moreover, chlorophyll content was measured with Dualex and no significant changes were observed in both experiments (Supplementary Figure S1, Supplementary Table S1).

### 2.2. Light-Induced Accumulation of UV-Absorbing Substances

**Figure 2:**
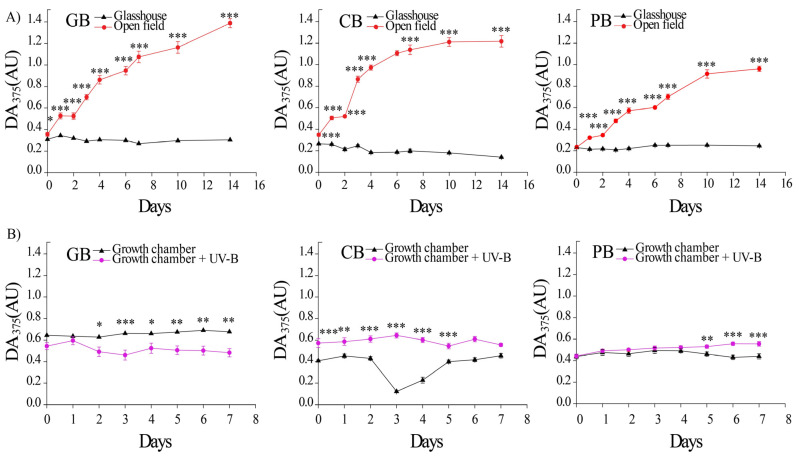
The dynamics of epidermal flavonoid (EpFlav) accumulation, in the leaves of three basil varieties (*Ocimum basilicum* var. Genovese—GB, *n* = 20; *Ocimum* × *citriodorum*—CB, *n* = 20; and *Ocimum basilicum* var. *purpurascens*—PB, *n* = 20) grown in (**A**) glasshouse (GH—black line) and open field (OF—red line) during 15 days; (**B**) growth chamber (GC—black line) and growth chamber with additional UV-B supplementation (UV-B—red line) during 8 days. Values are given as means ± SE. Significant differences in EpFlav between plants grown in GH and OF, as well as between plants grown in GC and UV-B are indicated for all varieties according to the Mann–Whitney U-test (* *p* ≤ 0.05, ** *p* < 0.01, *** *p* < 0.001).

In the glasshouse, the basal concentration of epidermal flavonoids (EpFlav, DA_375_) in the leaves of all three basil varieties ranged from 0.2 to 0.4 (AU). Nevertheless, an initial increase in EpFlav accumulation was detected 24 h after transferring the plants to the open field, and it continued to increase in all varieties, but the increase rate was the slowest in *Ocimum basilicum* var. *purpurascens* (Figure 2A). On the other hand, EpFlav content in leaves of plants grown continuously in the glasshouse remained constant. After 15 days, the content of epidermal UV-absorbing compounds was seven-fold higher in *Ocimum basilicum* var. Genovese and *Ocimum* × *citriodorum* and five-fold higher in *Ocimum basilicum* var. *purpurascens* grown in the open field compared to the respective varieties grown in the glasshouse.

Exposure to supplemental UV-B irradiation at constant PAR, comparable to the intensity of solar UV-B irradiation, resulted in only a slight increase in EpFlav accumulation in all three basil varieties, far less dramatic than in the open field conditions, where the EpFlav content increase was four–nine times higher in comparison to the glasshouse (Figure 2B, significant “light” effects, Supplementary Table S2).

### 2.3. Phenolic Profile of Basil Leaves under Different Light Regimes

Significantly higher basal levels of syringic acid (SyA), *p*-coumaric acid (*p*CA), and cyanidin (Cy) were found in the *Ocimum basilicum* var. *purpurascens* variety. Furthermore, anthocyanins were only detected in *Ocimum basilicum* var. *purpurascens*. *Ocimum basilicum* var. Genovese had significantly higher basal levels of hydroxycinnamic acids (HCAs), and the lowest basal levels of quercetin (Q), while *Ocimum* × *citriodorum* had the highest basal levels of protocatechuic acid (PrcA).

**Figure 3:**
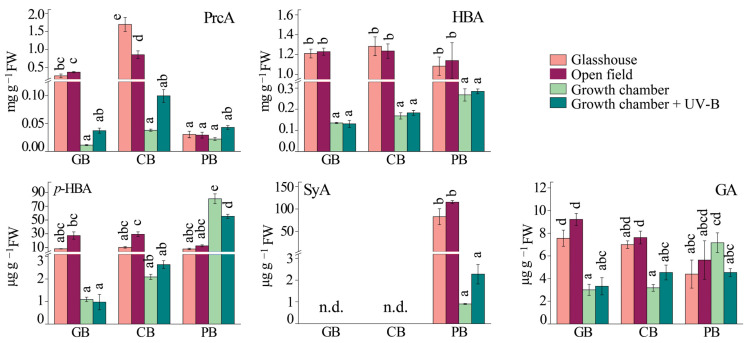
The content of hydroxybenzoic acids (HBAs) in the leaves of three basil varieties (*Ocimum basilicum* var. Genovese, GB, *n* = 7–9; *Ocimum* × *citriodorum*, CB, *n* = 7–9; and *Ocimum basilicum* var. *purpurascens*, PB, *n* = 7–9) grown under different conditions: glasshouse—GH (light purple), open field—OF (dark purple), growth chamber—GC (light green), and growth chamber + UV-B (dark green). Protocatechuic acid, PrcA; hydroxybenzoic acid, HBA; *p*-hydroxybenzoic acid, *p*-HBA; syringic acid, SyA; gallic acid, GA. Values are given as means ± SE. Different letters denote significant differences between the different treatments and basil varieties (n.d.—not detected, *p* ≤ 0.05), according to Tukey’s post hoc test.

Generally, the most abundant hydroxybenzoic acids (HBAs) in all three varieties under all light regimes were protocatechuic acid (PrcA) and unidentified HBA, followed by *p*-HBA, and gallic acid (GA) (Figure 3). The HBA and GA contents were not affected by full sunlight exposure or UV-B supplementation (Figure 3), while significant differences in the levels of PrcA and *p*-HBA induced with sunlight were observed between the three basil cultivars (Supplementary Table S3, ANOVA, *p* ≤ 0.05). The accumulation of *p*-HBA was slightly higher with full sunlight compared to the glasshouse for all three cultivars, while the highest accumulation of this compound was noted in the leaves of *Ocimum basilicum* var. *purpurascens* from the growth chamber. The content of syringic acid (SyA) (variety-specific for PB) was significantly higher in the growth chamber and the open field than in the glasshouse and under UV-B (Figure 3).

**Figure 4:**
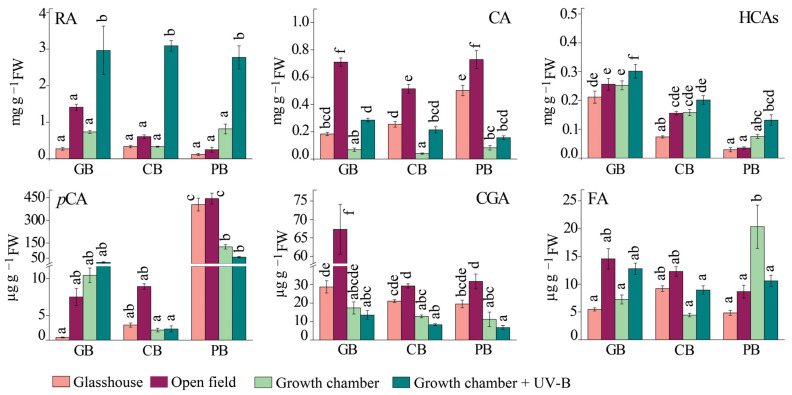
The content of hydroxycinnamic acids (HCA) in leaves of three basil varieties (*Ocimum basilicum* var. Genovese, GB, *n* = 7–9; *Ocimum* × *citriodorum*, CB, *n* = 7–9; and *Ocimum basilicum* var. *purpurascens*, PB, *n* = 7–9) grown under different conditions: glasshouse—GH (light purple), open field—OF (dark purple), growth chamber—GC (light green), and growth chamber + UV-B (dark green). Rosmarinic acid, RA; caffeic acid, CA; other hydroxycinnamic acids, HCAs; *p*-coumaric acid, *p*CA; chlorogenic acid, CGA; ferulic acid, FA. Values are given as means ± SE. Different letters denote significant differences between the different treatments and basil varieties (*p* ≤ 0.05), according to Tukey’s post hoc test.

The dominant hydroxycinnamic acids (HCA) in all three varieties were rosmarinic acid (RA), caffeic acid (CA), and *p*-coumaric acid (*p*CA). Sunlight exposure (open field) and UV-B supplementation had the most remarkable effects on the accumulation of RA in all three varieties, but to a greater extent with UV-B supplementation. Nevertheless, the content of RA within the same treatment was relatively similar among basil varieties (Figure 4, Supplementary Table S4, ANOVA, *p* ≤ 0.05).

Common responses to sunlight (OF) and UV-B supplementation were increased levels of caffeic acid (CA) and other unidentified hydroxycinnamic acids in all three cultivars. While full sunlight resulted in higher accumulation of CA in all three varieties (although the increase was the lowest in purple basil), UV-B supplementation had a much greater impact on total HCA content (the increase with UV-B treatment was from 5 to 9 µmol g^−1^ FW in *Ocimum basilicum* var. Genovese, 2.6 to 9.7 µmol g^−1^ FW in *Ocimum* × *citriodorum*, and 0.94 to 9 µmol g^−1^ FW in *Ocimum basilicum* var. *purpurascens*). Opposite were the effects of open field treatment compared to UV-B supplementation that were observed for chlorogenic acid (CGA) accumulation (Supplementary Table S4, Supplementary Figure S2). On the other hand, the content of ferulic acid (FA) was not significantly affected by the different treatments, although an increasing trend was observed in *Ocimum basilicum* var. Genovese in the open field (Figure 4, Supplementary Table S4, ANOVA, *p* ≤ 0.05).

**Figure 5:**
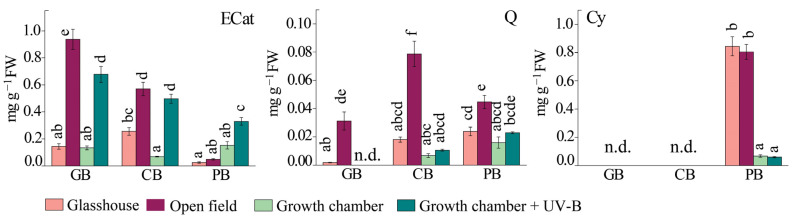
The content of epicatechin (ECat), quercetin (Q), and cyanidin (Cy) in the leaves of three basil varieties (*Ocimum basilicum* var. Genovese, GB, *n* = 7–9; *Ocimum* × *citriodorum*, CB, *n* = 7–9; and *Ocimum basilicum* var. *purpurascens*, PB, *n* = 7–9) grown under different conditions: glasshouse—GH (light purple), open field—OF (dark purple), growth chamber—GC (light green), and growth chamber + UV-B (dark green). Values are given as means ± SE. Different letters denote significant differences between the different treatments and basil varieties (*p* ≤ 0.05), according to Tukey’s post hoc test. n.d.—not detected.

Interestingly, the accumulation of flavan-3-ols, catechin (Cat), and epicatechin (ECat) was opposite in *Ocimum* × *citriodorum* and *Ocimum basilicum* var. *purpurascens* compared to the glasshouse and open field (Figure 5, Supplementary Table S5). ECat levels increased in *Ocimum* × *citriodorum* and *Ocimum basilicum* var. Genovese, whereas Cat levels were higher in *Ocimum basilicum* var. *purpurascens* and Cat was not found in *Ocimum basilicum* var. Genovese. Moreover, UV-B supplementation induced ECat accumulation in all three basil varieties compared with a respective treatment without UV-B (GC).

Furthermore, the induction of quercetin (Q) accumulation was a common response of all three varieties at full sunlight (OF), but to different extents: it increased almost 20-fold in *Ocimum basilicum* var. Genovese plants compared with the glasshouse plants, and 4-fold and 2-fold in *Ocimum* × *citriodorum* and *Ocimum basilicum* var. *purpurascens*, respectively (Figure 5, Supplementary Table S5). However, Q content in *Ocimum* × *citriodorum* and *Ocimum basilicum* var. *purpurascens* did not change significantly after UV-B exposure compared to the growth chamber in any other variety. As expected, the anthocyanin cyanidin (Cy) was only detected in *Ocimum basilicum* var. *purpurascens* leaves. Plants grown in the glasshouse and the open field had more than 10 times higher Cy content than plants grown in the growth chamber and under UV-B (Figure 5).

### 2.4. Antioxidant Response

**Figure 6:**
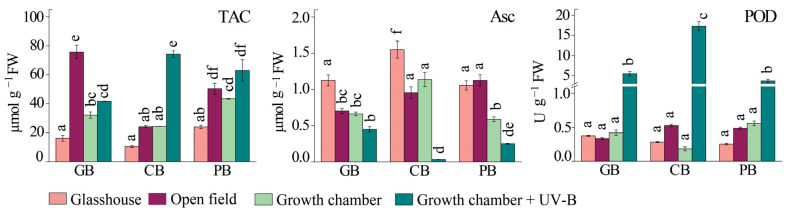
Total antioxidant capacity (TAC), reduced ascorbate (Asc) content, and class III peroxidases’ (PODs) activity of three basil varieties (*Ocimum basilicum* var. Genovese, GB, *n* = 7–9; *Ocimum* × *citriodorum*, CB, *n* = 7–9; and *Ocimum basilicum* var. *purpurascens*, PB, *n* = 7–9) grown under different conditions: glasshouse—GH (light purple), open field—OF (dark purple), growth chamber—GC (light green), and growth chamber + UV-B (dark green). Values are given as means ± SE. Different letters denote significant differences between the different treatments and basil varieties (*p* ≤ 0.05), according to Tukey’s post hoc test.

Total antioxidant capacity (TAC), as determined with the ABTS assay, was highest in the leaves of *Ocimum basilicum* var. *purpurascens* plants under glasshouse and growth chamber light conditions (Figure 6). When plants were exposed to full sunlight for 15 days (open field), TAC increased in all three basil varieties compared to plants grown in the glasshouse. The highest increase was measured in *Ocimum basilicum* var. Genovese (about 4.6-fold higher TAC values compared with the glasshouse), followed by *Ocimum* × *citriodorum* and *Ocimum basilicum* var. *purpurascens* (about 2-fold higher values) (Figure 6). In contrast, exposure to artificial UV-B irradiation induced only a slight increase in TAC in *Ocimum basilicum* var. Genovese and *Ocimum basilicum* var. *purpurascens*, whereas a three-fold increase was observed in *Ocimum* × *citriodorum* (Supplementary Table S6, ANOVA, *p* ≤ 0.05).

The concentration of reduced ascorbate (Asc) was decreased in *Ocimum basilicum* var. Genovese and *Ocimum* × *citriodorum* plants grown in the open field compared to those grown in the glasshouse. At the same time, no significant changes in reduced ascorbate content were observed in *Ocimum basilicum* var. *purpurascens* plants when the plants were exposed to full sunlight. Moreover, the content of reduced ascorbate was significantly decreased in all plants under UV-B treatment in contrast to the control plants in the growth chamber (Figure 6).

No significant changes in the activity of the class III peroxidases (PODs) were observed in the plants grown in the open field compared to plants grown in the glasshouse (Figure 6, Supplementary Table S6). POD activity was similar between basil varieties, as well as between the different treatments. On the other hand, the POD activity drastically increased in response to UV-B supplementation in all three varieties compared to the growth chamber.

### 2.5. H_2_O_2_ Accumulation in Leaves

**Figure 7:**
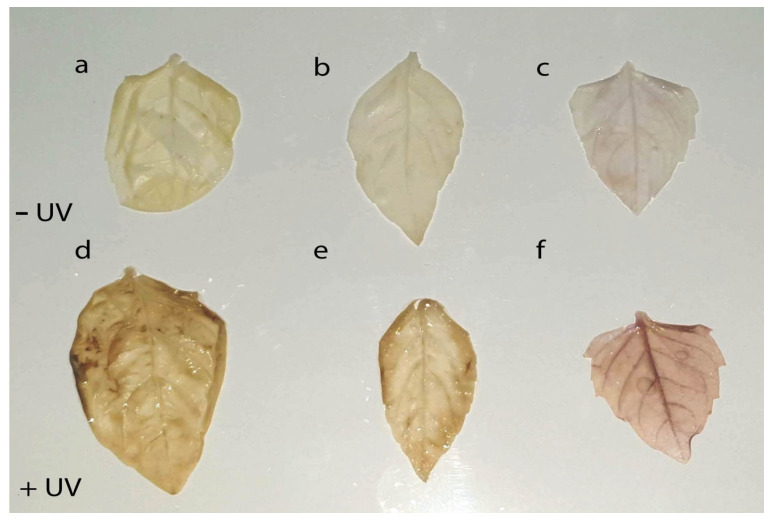
Representative images of H_2_O_2_ accumulation using the DAB uptake method in the leaves of three basil varieties grown at constant PAR (**a**–**c**) and constant PAR with artificial UV-B irradiation (**d**–**f**) after 8 days of treatment. *Ocimum basilicum* var. Genovese (GB) (**a**,**d**), *Ocimum* × *citriodorum* (CB) (**b**,**e**), and *Ocimum basilicum* var. *purpurascens* (PB) (**c**,**f**).

Visible signs of damage in response to changing light conditions were observed only in experiment II, following exposure to UV-B irradiation at constant PAR. To elucidate whether UV-B irradiation provoked oxidative injury in leaves, 8 days after the start of treatment, H_2_O_2_ accumulation was visualized with the DAB uptake method. Indeed, in all three basil varieties, an increased H_2_O_2_ accumulation was observed both in the veins and in mesophyll cells compared to leaves of non-UV-B treated plants (Figure 7).

## 3. Discussion

### 3.1. Contrasting Effects of Sunlight and UV-B/Moderate Light

The three varieties of basil used in the study showed specific, as well as general, responses to changing light conditions—full sunlight or UV-B supplementation under moderate PAR. While high sunlight including natural UV-B irradiation developed no visible signs of photo-oxidative damage in any variety, it induced a strong antioxidant response with a significant increase in TAC and total HCAs and both total flavonoid leaf content and epidermal flavonoids indicated ROS signaling in antioxidant defense [40]. In contrast, a supplemental UV-B irradiation (daily dose: 29.16 kJ m^−2^ d^−1^, comparable to the intensities of ambient UV-B) combined with moderate background PAR induced oxidative stress within 8 days, which may imply a deficient mechanism of antioxidative defense under the given condition [11]. This was demonstrated with the accumulation of H_2_O_2_ and the appearance of brown lesions on the upper surface of mature leaves. Moreover, under supplemental UV-B irradiation, the formation of the UV-absorbing shield failed, which was accompanied by the absence of quercetin accumulation, indicating its important role in UV-B protection and the possible role of UV-A or high PAR in its formation. The results are not in agreement with numerous reports highlighting the central role of UV-B radiation in induction of the epidermal UV shield in a variety of plant species [2,41,42,43]. Interestingly, the high efficiency of UV-B radiation in promoting flavonoid biosynthesis has been demonstrated even at very low UV-B fluence rates [44,45,46]. The failure of UV-B/moderate light to induce epidermal flavonoids’ accumulation could be explained with the lack of interaction with COP1, which can be triggered with an excess of PAR or blue light that upregulates chalcone synthase, a key enzyme for flavonoid biosynthesis, via the COP1/HY5 pathway [44,47]. Preferential accumulation of phenolics under high light over UV was observed in *Pelargonium zonale* leaves [48]. In addition, supplemental UV-B induced a change in the morphology of developing leaves similar to stress-induced morphogenic responses (SIMRs) commonly induced with high UV-B radiation and ROS accumulation [26,49]. UV-B-induced oxidative stress has been characterized as a non-specific response to ROS generated with high UV-B radiation [50]. The simultaneous drastic increase in PODs’ activity and decrease in reduced Asc content in leaves under supplemental UV-B indicated the predominant pro-oxidative effect of UV-B irradiation (indicated with H_2_O_2_ accumulation in the leaf) and the strong antioxidant response of plants [51]. Instead, acclimation to sunlight resulted in leaf thickening, indicating a possible important role of the induced formation of a secondary cell wall, which led to an almost two-fold decrease in the FW/DW biomass ratio, in addition to the known protective formation of an epidermal UV shield of UV-absorbing substances. Similar effects of low UV-B radiation have been reported by other authors [26,52]. However, in addition to the fluence rate of UV-B; the ratio between the UV-B and background PAR intensity, blue light, and UV-A radiation; as well as other climate factors may determine the contradictory UV-B effects on plants, i.e., stress response or regulation [41,43,44]. Therefore, there is a possibility that UV-A radiation could have beneficial effects on plant acclimation with a shifted ratio of UV-B/PAR; however, this remains to be explored. In our study, the stimulation of phenylpropanoid metabolism, but different classes of phenolics, in relation to different UV-B/PAR ratios, suggests the importance of HCAs and flavonoids in antioxidative defense against photo-oxidation.

### 3.2. The Epidermal UV-Attenuation Capacity in Green vs. Purple Leaves

The most striking difference between the varieties was visible in the dynamics of accumulation of epidermal UV-attenuating compounds upon exposure to the open field conditions. The maximum levels of EpFlav were reached after 11 days, with a delay of 2 to 3 days before the first significant increase. Previously, we observed that the induction period to reach maximal EpFlav levels upon exposure to full sunlight is species-specific and is reached earlier in *Solanum lycopersicum*, *Salvia officinalis*, and *Eruca sativa* compared to basil plants in this study, after 4 days [53].

Similar dynamics in the formation of the protective sun-screening shield have also been reported for other plant species [42,54]. However, the mechanism underlying the delay in EpFlav accumulation has not yet been elucidated. Bidel and co-authors (2015) suggested that the observed kinetics of plant response to a new light regime might depend on the combination of environmental factors, as well as on the previous plant’s history [42]. In their comprehensive study of UV-B-induced dynamics of EpFlav accumulation in *Centella aciatica*, the authors suggested that the screening effect may contribute to a slower accumulation of flavonoids as a result of negative feedback regulation of flavonol biosynthesis due to exponential attenuation of UV-B radiation through the epidermis [42]. The discrepancy between the high sensitivity and rapid activation of the UVR8 signaling pathway [55,56] on the one hand and the slow attainment of maximal EpFlav on the other hand could be explained with an interplay between UV-B radiation and other specific phenylpropanoid inducers such as UV-A, blue light, or high PAR fluxes in general [57]. The higher basal accumulation of anthocyanins, syringic acid, *p*-coumaric acid, and caffeic acid in *Ocimum basilicum* var. *purpurascens* may be the reason for the slower response to full light exposure. The anthocyanins in red basil leaves are primarily located in the abaxial and adaxial epidermises of basil leaves [58]. Thus, they can preferentially serve as partial absorbers of incident light energy, slowing the generation of ROS with the excess light-exposed chloroplasts. On the other hand, it had the highest chlorophyll concentration in the open field towards the later acclimation phase compared to the other two varieties, as observed previously [36]. Comparative study of full sunlight acclimation of green and purple basil leaves showed that purple leaves have shade avoidance characteristics and that high-light-responsive genes are downregulated [36].

### 3.3. Accumulation of Asc, HCAs, and Flavonoids Is Affected by the Different UV-B/PAR Ratio

Under both sunlight and supplemental UV-B irradiation, TAC was enhanced in the leaf extract of all three varieties; strikingly, the highest TAC increase occurred in *Ocimum* × *citriodorum* under both light regimes. On the other hand, UV-B decreased the level of reduced ascorbate compared with sunlight. Although the leaf constitutive molar content of ascorbate exceeded that of total phenolics by more than 500-fold, the accumulated HCAs and flavonoids contributed more to TAC under stress conditions [21]. Among the HCAs, RA was most abundant in *Ocimum basilicum* var. *purpurascens* (74% and 90% of all HCAs under open field conditions, and UV-B, respectively) and least abundant in *Ocimum basilicum* var. Genovese (64% and 82% of all HCAs at open field, and UV-B, respectively). We measured the antioxidant capacity of each compound present in basil leaves. Their activity decreased in descending order, RA > Cat > ECat > CGA > Q > Cy > FA > CA > Eriodictyol > Asc (Supplementary Table S7), and this is in accordance with Rice-Evans et al. (1997) [21]. Full sunlight exposure (a combination of UV radiation and high PAR intensity) can lead to increased oxidative stress. In our study, it led to a decrease in the ratio of Asc/HCAs (flavonoids), indicating carbon allocation from primary to secondary metabolism, towards the biosynthesis of phenolics and growth inhibition (Supplementary Figure S3).

We propose that induced hydroxycinnamic acids (HCAs), flavonoids, and cyanidin may play a role in H_2_O_2_ scavenging via PODs. The resulting phenoxyl radical can be reduced by ascorbate in the so-called phenolics/peroxidase/ascorbate cycle (PPA), which is localized in the apoplast and vacuole, as proposed by Takahama and Oniki (1997) [51]. However, the ratio between HCAs and flavonoids was also changed: in comparison to sunlight (open field including ambient UV-B and UV-A), UV-B radiation supplemented to PAR increased the HCAs/flavonoids ratio in all three basil varieties. The role of HCAs in response mechanisms to excess UV radiation has been underestimated in previous studies [59]. Tattini and co-authors (2004) reported differential spatial accumulation of these two polyphenol groups (flavonoids and HCAs) under excess light and drought [60]. They proposed that flavonoids play a preferential screening role in the epidermis, while HCAs act as important antioxidants in palisade leaf tissue. However, HCAs’ absorbance characteristics imply even greater protection than flavonoids against UV-B-induced damage [61]. Thus, HCAs might be crucial components in the acclimation mechanisms to full solar radiation under field conditions [59,62].

### 3.4. Differential Photomorphogenic and Antioxidative Response of Three Basil Varieties

The obtained results indicate that UV-B radiation under moderate PAR does not cause significant accumulation of flavonoids in the epidermis, but instead induces morphogenic changes, especially in younger leaves. The most sensitive variety, GB with visible lesions, browning, and vitrification of the upper exposed leaf surface, had the lowest TAC and no epidermal flavonoid accumulation under artificial UV-B irradiation, accompanied by no significant increase in quercetin.

In *Ocimum basilicum* var. *purpurascens* and *Ocimum* × *citriodorum*, DW increased almost two-fold at OF, which may indicate enhanced content of cell wall compounds (e.g., lignin). Increased lignification in response to UV-B radiation has been observed elsewhere [26,63]. HCAs are structural components of the cell wall and provide mechanical reinforcement of the cell wall through the formation of covalent cross-links with lignin and polysaccharides.

The extent of the antioxidant responses described above depended on the basil variety, in descending order: *Ocimum* × *citriodorum* > *Ocimum basilicum* var. Genovese > *Ocimum basilicum* var. *purpurascens*. Furthermore, full sunlight had no effect on both the reduced ascorbate level and POD activity of *Ocimum basilicum* var. *purpurascens*, which is consistent with the higher constitutive antioxidative capacity of anthocyanins [64]. A common response in all three basil varieties was the induction of rosmarinic acid (RA) and caffeic acid, under supplementary UV-B irradiation and open field conditions, upon transferring plants, though the induction was smaller in open field conditions. Based on the scavenging capacity of the ABTS radicals, RA was the most potent antioxidant among the phenolics found in basil leaves. Cyanic leaves of *Ocimum basilicum* var. *purpurascens* had the lowest content of RA, and the light effect on the biosynthesis of RA was the least pronounced, suggesting that this compound was not the main component of acclimation in this variety. Cyanidin, on the other hand, remained unchanged in the *Ocimum basilicum* var. *purpurascens* variety regardless of light conditions. In addition, the slower accumulation of epidermal flavonoids in sunlight in this variety compared to *Ocimum* × *citriodorum* and *Ocimum basilicum* var. Genovese may be attributed to a higher shielding effect of the cyanic leaves [65].

The variety-specific differences not only in the dynamics of epidermal flavonoid induction, but also in the level of reduced ascorbate (reduced in green but not in purple leaves) upon open field acclimation, and in the profile of total phenolics, are important for the selection of the best variety for growers in relation to regional conditions and for the choice of the optimal harvest time to achieve the best functional quality of basil.

## 4. Materials and Methods

### 4.1. Plant Material and Growth Conditions

Seeds of *Ocimum basilicum* var. Genovese (GB), *Ocimum* × *citriodorum* (hybrid of *Ocimum basilicum* and *Ocimum americanum*, CB), and *Ocimum basilicum* var. *purpurascens* (‘Red Rubrum’, PB) were obtained commercially and sown in the substrate Klasman-Potgrond H (Klasmann-Deilmann, Geeste, Germany). Plants were grown in different systems: in a glasshouse (GH), in the open field (OF), and in a growth chamber without UV-B supplementation (GC) and with UV-B supplementation (UV-B).

The first part of the experiments started in spring, when basil was grown in the GH. Two-week-old uniform plants were randomly divided and planted in 8 × 8 cm plastic pots. The seedlings were grown for another 2 weeks in the GH without additional lighting or heating until they reached a height of about 4–5 cm. Then, they were repotted into larger pots and grown for the next 3 weeks until they reached the stage of 4 fully developed leaves and a height of about 15–20 cm.

The basil plants obtained were divided into two groups: the control group, which remained in the GH, and group OF, which was exposed to full sunlight for the rest of the treatment (15 days in total). Radiation conditions (PAR, UV-A, and UV-B) were measured daily in both growing systems. The intensity of PAR was measured with the PAR Quantum Sensor CE (SKP 215 42474; Skye Instruments, Llandrindod Wells, Powys LD1 6DF, UK), whereas intensity of UV-A radiation was measured with the PMA 2100 radiometer (Solar Light Company Inc., Glenside, PA, USA), equipped with UV-A (PMA 2110, 320–400 nm). A UV-B detector for biologically active radiation (Solar Light’s Model 501 Series Biometer-Radiometers), which has continuously measured the UV index over Belgrade since 2009 [66], was used to assess UV-B radiation using the McKenzie and co-authors’ [67] relationship between erythemally weighted UV-B and UV-B_280–315 nm_. The average daily temperature in the GH and field was 22.0 ± 0.8 °C and 25.2 ± 4.1 °C, respectively, while the relative humidity was 30–40% in the glasshouse and 40–50% in the OF.

For the second part of the experiment, the plants were grown in GC under controlled conditions with a photoperiod of 14/10 h (day/night), a temperature of 26 °C, and a relative humidity of 40–50%. The plants were grown as described for the first part of the experiment. Half of the plantlets were exposed to supplemental UV-B irradiation (1.35 W m^−2^) for 6 h daily, for 8 days (29.16 kJ m^−2^ d^−1^). For UV-B supplementation, a UV lamp with a visible light philter (Carl Roth GmbH, Karlsruhe, Germany; product number: H469.1) and tubes with maximum emission at 312 nm (G15T8E, Sankyo Denki, Tokyo, Japan) were used. Lamp radiation intensity was characterized using the Vilber Lourmat instrument with the CX-312 sensor (312 nm medium-wave UV, bandwidth: 280 to 320 nm). The intensity of irradiation with the UV-B lamp was determined with the distance from the surface of the plant leaves (approx. 0.45 m), while the daily energy of UV-B radiation was chosen to correspond with the previously performed OF measurements.

The content of chlorophyll (Chl), leaf epidermal flavonoids (EpFlav), and the nitrogen balance index (NBI), based on the Chl/flavonoids ratio, were measured daily using the Dualex 4 (FORCE-A, Orsay, France; Cerović et al., 2012) [68]. All measurements were performed on healthy, fully developed, and light-exposed leaves.

Four to five biological replicates of all three genotypes were used for each light treatment (GH, OF, GC, and UV-B). For biochemical measurements, light exposed, fully developed leaves were harvested (three to four leaves per plant, 4–5 plants per treatment). Leaf material was immediately frozen in liquid nitrogen and stored at −80 °C for further analyses. The remaining plants from GH and OF were used to determine fresh weight and dry weight of leaves (*n* = 4–5). To obtain the dry weight, samples were dried at 70 °C for 48 h.

### 4.2. Phenolics Determination with HPLC-DAD

Frozen leaves were rapidly homogenized in liquid nitrogen and extracted in methanol containing 0.1% HCl, followed by acid hydrolysis to obtain aglycones. All extracts were purged with nitrogen and stored at −80 °C until a further analysis [43].

The phenolic compounds were identified and quantified with HPLC-PDA (LC-20AB Prominence liquid chromatography, Shimadzu, Kyoto, Japan) with an SPD-M20A diode array Prominence detector using a reversed-phase C18 column (5.0 µm, 250 × 4.6 mm Luna C18 (2); Phenomenex Ltd., Torrance, CA, USA). The elution procedure was 0–5 min, 100% solution B (isocratic step); 5–25 min, 100–80% solution B (linear gradient); 25–35 min, 80–60% solution B (linear gradient); 35–40 min, 60–100% solution B (linear gradient). Chromatograms were recorded at different wavelengths, depending on the characteristic absorption maximum of the selected phenolics: 320 nm for hydroxycinnamic acids and their derivatives, 280 nm for catechins, hydroxybenzoic acids, and their derivatives, and 520 nm for anthocyanins. The individual phenolics were identified by comparing the absorption spectra with authentic standards and quantified with peak area using Shimadzu LC Solution software (LCsolution Version1.25 SP2, Shimadzu, Kyoto, Japan) [43].

### 4.3. Measurement of Reduced Ascorbate

For the determination of reduced ascorbate content, frozen leaf samples were rapidly homogenized in liquid nitrogen, extracted in 1.5% meta-phosphoric acid containing 1 mM EDTA, and centrifuged for 5 min at 16,000× *g* and 4 °C, according to Morina et al. (2010) [69]. The reduced form of ascorbate was analyzed with the decrease in absorbance at 265 nm after the addition of one unit of ascorbate oxidase (Sigma Aldrich, Steinheim am Albuch, Germany) in reaction mixtures consisting of 950 µL of a 50 mM sodium phosphate buffer (pH 6.5) containing 2.5 mM EDTA and 50 µL of a plant extract (ε = 14.3 mM^−1^ cm^−1^) [69].

### 4.4. Determination of POD Activity

For soluble class III peroxidases (PODs, EC 1.11.1.7), frozen leaf material was homogenized in liquid nitrogen and extracted in a 100 mM sodium phosphate buffer (pH 6.5) with 2 mM EDTA, 2 mM PMSF (phenylmethanesulfonyl fluoride), and 5% (*w*/*v*) insoluble polyvinylpyrrolidine (PVP). The homogenate was centrifuged at 10,000× *g* for 10 min at 4 °C. The peroxidase activity of POD was measured spectrophotometrically in a reaction mixture of a 100 mM K-phosphate buffer (pH 6.5), 20 mM guaiacol, and aliquots of the plant extract [43]. The reaction was started by adding 5 mM H_2_O_2_, and the increase in absorbance at 470 nm was followed. Peroxidase activity was calculated using the extinction coefficient for guaiacol (ε = 26.6 mM^−1^ cm^−1^).

### 4.5. Antioxidant Capacity

The methanol leaf extracts described in Section 4.3. were used for the determination of total antioxidative capacity (TAC). The reaction mixture contained 2 mM 2,2′-azino-bis(3-ethylbenzothiazoline-6-sulfonic acid) (ABTS), 0.015 mM H_2_O_2_, and horseradish peroxidase (HRP) in a 50 mM potassium phosphate buffer (pH 7.5) and 20 μL of the extract. The absorbance was measured at 730 nm. The ascorbic acid standard curve was used to determine the relative antioxidant capacity of the samples [70].

### 4.6. Determination (Visualization) of H_2_O_2_ in Basil Leaves

To detect H_2_O_2_ accumulation, three sun-exposed, fully developed leaves per plant (three plants per group) were cut from GC and UV-B treatments and incubated with 3,3′-diaminobenzidine (DAB) [71]. Leaves were placed in 1 mg mL^−1^ (DAB-HCl) in a 100 mM sodium acetate buffer (pH 3.8), infiltrated for 15 s at low vacuum, and incubated for 2 h with shaking. Leaves were cleared in boiling 70% (*v/v*) ethanol (for 10 min) with 10% glycerol.

### 4.7. Statistical Analysis

A one-way repeated-measures ANOVA was conducted to determine differences in EpFlav and Chl content during the experiment (within-subject factor) in plants exposed to different treatments (between-subject factors). To test for significant differences in FW, DW, FW/DW, EpFlav, and Chl, content between the different treatments, the Mann–Whitney *U*-test was used, and the significance threshold was set at 0.05. Two-way ANOVA was used to show the effects of variety type, treatment, and their interaction on the content of phenolic compounds, TAC, reduced ascorbate content, and POD activity. The homogeneity of variance was checked with Levene’s test. The experimental data were analyzed using the software package Statistica 8.0.

## 5. Conclusions

In this study, we demonstrated a contrasting effect of UV-B radiation on the leaves of three basil varieties depending on the quality and intensity of background light. Different UV-B/UV-A/PAR ratios affected (1) epidermal UV shielding capacity; (2) Asc/HCAs/flavonoids ratio; and (3) antioxidant response in three basil varieties, which may imply the variety-specific tolerance to high PAR and UV-B irradiation. We have shown here that full sunlight overcomes the limitations of supplemental artificial UV-B radiation in combination with moderate PAR to induce the formation of a UV-absorbing shield in the basil leaf based on the accumulation of flavonoids, particularly quercetin. Under full sunlight, the potential risk of UV-B-induced photo-oxidative stress in the leaves of three basil varieties was suppressed. While the response to UV-B-induced photo-oxidation differed among varieties in terms of magnitude and defense strategy, the response to full sunlight was more general, similar among all selected varieties and involved the increase in epidermal flavonoid accumulation. The most striking difference in the responses of the three basil varieties to UV-B is the POD induction and the possible involvement of the PPA cycle, which is greatest in CB and lowest in PB with constitutively high cyanidin content. Thus, the results of our study provided further evidence for the effects of epidermal anthocyanins and flavonoids, as potent antioxidants and philters of PAR and UV-B radiation on the morphological and antioxidant protection responsible for the different acclimation mechanisms to high solar irradiance and UV-B irradiation in red and green leaves. We have shown that purple-leaf basil directs lower assimilated carbon flux to other non-red phenylpropanoids (quercetin, epicatechin) compared to green basil varieties at full sunlight. This indicates competition between flavonol and anthocyanin biosynthesis, possibly leading to two defense strategies in green and purple varieties. We suggest that the role of the philter and sink for reduced carbon in the purple-leaf variety is more responsible for the specific tolerance mechanism in this variety, implying a lower excitation pressure in its leaves. On the other hand, hydroxycinnamates and PODs were found to be the main protective mechanism against UV-B, particularly in the green cultivar CB.

## Figures and Tables

**Table 1:** Experiment I. Leaf fresh weight (FW) and dry weight (DW) (g) and their ratio after 15 days of treatment in the open field exposed to full sunlight (OF) and continuous treatment in the glasshouse (GH). Values represent the sum of the masses of all leaves from individual plant; means ± SE (*n* ≥ 4). Significant differences between GH and OF plants according to *t*-test are indicated (* *p* ≤ 0.05, ** *p* < 0.01, *** *p* < 0.001). *Ocimum basilicum* var. Genovese (GB), *Ocimum* × *citriodorum* (CB), and *Ocimum basilicum* var. *purpurascens* (PB).

	GB		CB		PB	
	GH	OP	GH	OP	GH	OP
**FW (g)**	19.00 ± 1.78	12.33 ± 0.62 *	8.67 ± 0.94	12.00 ± 1.47	9.00 ± 0.82	8.67 ± 0.94
**DW (g)**	1.56 ± 0.13	1.80 ± 0.08	0.86 ± 0.05	1.65 ± 0.21 *	0.53 ± 0.02	0.91 ± 0.06
**FW/DW**	12.18 ± 0.17	6.88 ± 0.36 ***	10.16 ± 0.39	7.27 ± 0.06 **	16.88 ± 0.97	9.47 ± 0.47 **

## Data Availability

Not applicable.

## Supplementary Materials

The supporting information can be downloaded at: https://www.mdpi.com/article/10.3390/ijms242015350/s1.
