# Supplementary material for: Differential Antioxidant Response to Supplemental UV-B Irradiation and Sunlight in Three Basil Varieties
Source: Int J Mol Sci. 2023 Oct 19;24(20):15350. doi: 10.3390/ijms242015350 (PMC10607338; doi:10.3390/ijms242015350)
Supplement: Supplementary file 1 [file ijms-24-15350-s001.zip › ijms-2652761-supplementary.pdf]

Supplementary Materials

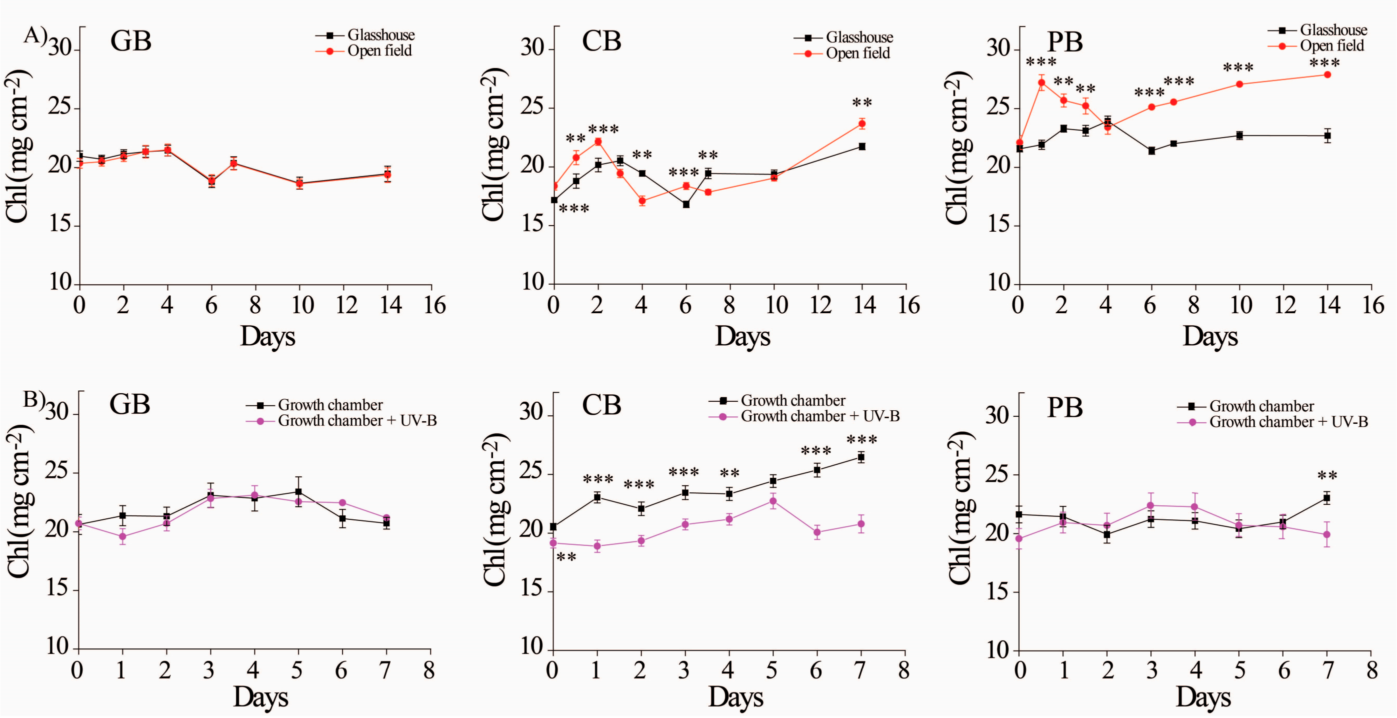

Figure S1. The dynamics of chlorophyll (mg cm^{-2}) accumulation in the leaves of three basil varieties (*Ocimum basilicum* var. Genovese – GB, *n* = 20; *Ocimum* × *citriodorum* – CB, *n* = 20 and *Ocimum basilicum* var. *purpurascens* – PB, *n* = 20) grown in (**A**) glasshouse (GH – black line) and open field (OF – red line) during 15 days; (**B**) growth chamber (GC – black line) and growth chamber with additional UV-B supplementation (UV-B – red line) during 8 days. Values are given as means ± SE (* $p \leq 0.05$, ** $p < 0.01$, *** $p < 0.001$). Significant differences in EpFlav between plants grown in GH and OF, as well as between plants grown in GC and UV-B for all varieties according to Mann–Whitney U/-test are indicated.

Table S1. Repeated-measures analyses of variance (profile analyses) on chlorophyll accumulation determined during 16 (glasshouse – GH and open field – OF) and 8 days (growth chamber – GC and growth chamber + UV-B – UV-B) of the of three basil varieties, *Ocimum basilicum* var. Genovese (GB), *Ocimum* × *citriodorum* (CB) and *Ocimum basilicum* var. *purpurascens* (PB).

Trait	GH-OF, source	*Df*	*F*	*P* > *F*	Trait	GC-UV-B, source	*Df*	*F*	*P* > *F*
	Between-subjects	1;38				*Between-subjects*	1;57		
	Light		0.182	0.67		Light		0.61	0.44
**GB**	*Within-subjects*	1;7			**GB**	*Within-subjects*	1;7		
	Day		12.26	<0.0001		Day		6.34	<0.0001
	Day × Light		0.142	<0.0001		Day × Light		1.16	0.33
	Between-subjects	1;38				*Between-subjects*	1;60		
	Light		0.77	0.38		Light		53.41	<0.0001
**CB**	*Within-subjects*	1;7			**CB**	*Within-subjects*	1;7		
	Day		41.33	<0.0001		Day		16.52	<0.0001
	Day × LIght		20.59	<0.0001		Day × LIght		4.92	<0.0001
	Between-subjects	1;38				*Between-subjects*	1;60		
	Light		22.06	<0.0001		Light		14.10	0.18
**PB**	*Within-subjects*	1;8			**PB**	*Within-subjects*	1;7		
	Day		1603.56	<0.0001		Day		1.33	0.25
	Day × LIght		25.00	<0.0001		Day × LIght		2.53	0.03

Table S2. Repeated-measures analyses of variance (profile analyses) on epidermal flavonoids (EpFlav) accumulation determined during 16 (glasshouse – GH and open field – OF) and 8 days (growth chamber – GC and growth chamber + UV-B – UV-B) of the of three basil varieties, *Ocimum basilicum* var. Genovese (GB), *Ocimum* × *citriodorum* (CB) and *Ocimum basilicum* var. *purpurascens* (PB).

Trait	GH-OF, source	*Df*	*F*	*P* > *F*	Trait	GC-UV-B, source	*Df*	*F*	*P* > *F*
	Between-subjects	1;38				*Between-subjects*	1;57		
	Light		1382.52	<0.0001		Light		12.294	0.001
**GB**	*Within-subjects*	1;8			**GB**	*Within-subjects*	1;7		
	Day		66.57	<0.0001		Day		0.49	0.75
	Day × LIght		75.32	<0.0001		Day × LIght		1.06	0.38
	Between-subjects	1;38				*Between-subjects*	1;60		
	Light		582.85	<0.0001		Light		185.53	<0.0001
**CB**	*Within-subjects*	1;7			**CB**	*Within-subjects*	1;7		
	Day		337.23	<0.0001		Day		11.54	<0.0001
	Day × LIght		479.57	<0.0001		Day × LIght		24.01	<0.0001
	Between-subjects	1;38				*Between-subjects*			
	Light		402.78	<0.0001		Light		15.31	<0.0001
**PB**	*Within-subjects*	1;7			**PB**	*Within-subjects*	1;7		
	Day		236.93	<0.0001		Day		2.63	0.02
	Day × LIght		188.29	<0.0001		Day × LIght		3.09	0.01

Table S3. Two-way ANOVA results for the effects of three varieties, *O. basilicum* var. Genovese (GB), *O.* × *citriodorum* (CB) and *O. basilicum* var. *purpurascens* (PB), and different light conditions, as well as their interaction on the content of hydroxybenzoic acids (HBAs) in the basil leaves. The *Dfs* are shown in the brackets (the first number represents the *Df* of the main effects and their interactions and the second number is *Df* of error).

Trait	Source of variation	*F*	*P* > *F*	Trait	Source of variation	*F*	*P* > *F*
**PrcA**	Variety	257.21	0	**HBAs**	Variety	0.57	0.57
	Light	122.3	0		Light	341.41	0
**(*Df* : 1;86)**	Variety × Light	93.08	0	**(*Df* : 1;85)**	Variety × Light	2.97	0.01
***p*-HBA**	Variety	55.72	<0.0001	**GA**	Variety	0.15	0.86
	Light	8.08	<0.0001		Light	10.29	<0.0001
**(*Df* : 1;85)**	Variety × Light	34.85	0	**(*Df* : 1;90)**	Variety × Light	6.37	<0.0001

Table S4. Two-way ANOVA results for the effects of three varieties, *Ocimum basilicum* var. Genovese (GB), *Ocimum* × *citriodorum* (CB) and *Ocimum basilicum* var. *purpurascens* (PB), and different light conditions, as well as their interaction on the content of hydroxycinnamic acids (HCAs) in the basil leaves. The *Dfs* are shown in the brackets (the first number represents the *Df* of the main effects and their interactions and the second number is *Df* of error).

Trait	Source of variation	*F*	*P* > *F*	Trait	Source of variation	*F*	*P* > *F*
**RA**	Variety	1.63	0.2	**CA**	Variety	19.12	0
	Light	72.89	0		Light	204.79	0
**(*Df* : 1;86)**	Variety × Light	1.92	0.87	**(*Df* : 1;85)**	Variety × Light	14.94	0
**HCAs**	Variety	38.43	<0.0001	**pCA**	Variety	461.35	0
	Light	9.26	<0.0001		Light	54.85	0
**(*Df* : 1;85)**	Variety × Light	0.27	0.95	**(*Df* : 1;90)**	Variety × Light	71	0
**CGA**	Variety	28.43	<0.0001	**FA**	Variety	1.02	0.36
	Light	73.57	0		Light	2.09	0.11
**(*Df* : 1;90)**	Variety × Light	7.57	<0.0001	**(*Df* : 1;89)**	Variety × Light	6.19	<0.0001
**SA**	Variety	8.31	<0.001				
	Light	26.21	<0.0001				
**(*Df* : 1;81)**	Variety × Light	3.96	0.002				

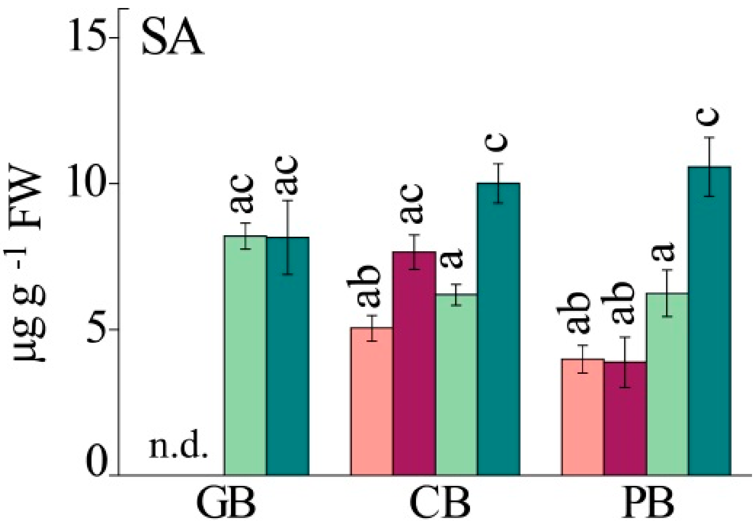

Figure S2. Content of sinapinic acid (SA) in the leaves of three basil varietes (*Ocimum basilicum* var. Genovese, GB, *Ocimum × citriodorum*, CB and *Ocimum basilicum* var. *purpurascens*, PB) grown under different conditions: glasshouse – GH (light purple), open field – OF (dark purple), growth chamber – GC (light green) and growth chamber + UV-B – UV-B (dark green)). Values are presented as means ± SE (n = 7–9). Different letters denote significant differences between different light conditions and basil varietes ($p \le 0.05$), according to Tukey's post hoc test. n.d.- not detected.

Table S5. Two-way ANOVA results for the effects of three varieties, *Ocimum basilicum* var. Genovese (GB), *Ocimum × citriodorum* (CB) and *Ocimum basilicum* var. *purpurascens* (PB), and different light conditions, as well as their interaction on the content of ECat, Cat, Q and Cy in the basil leaves. The *Dfs* are shown in the brackets (the first number represents the *Df* of the main effects and their interactions and the second number is *Df* of error).

Trait	Source of variation	*F*	*P* > *F*	Trait	Source of variation	*F*	*P* > *F*
**ECat**	Variety	257.21	0	**Cat**	Variety	227.32	0
	Light	122.3	0		Light	274.76	0
**(*Df* : 1;85)**	Variety × Light	93.08	0	**(*Df* : 1;77)**	Variety × Light	84.51	0
**Q**	Variety	28.03	0				
	Light	45.09	0				
**(*Df* : 1;40)**	Variety × Light	20.62	0				

Table S6. Two-way ANOVA results for the effects of three varieties, *Ocimum basilicum* var. Genovese (GB), *Ocimum × citriodorum* (CB) and *Ocimum basilicum* var. *purpurascens* (PB), and different light conditions, as well as their interaction on the content of TAC, Asc content and POD activity in the basil leaves. The *Dfs* are shown in the brackets (the first number represents the *Df* of the main effects and their interactions and the second number is *Df* of error)..

Trait	Source of variation	*F*	*P* > *F*	Trait	Source of variation	*F*	*P* > *F*
**TAC**	Variety	16.33	<0.0001	**Asc**	Variety	9.69	<0.001
	Light	117.63	0		Light	124.93	0
**(*Df* : 1;36)**	Variety × Light	34.08	<0.0001	**(*Df* : 1;36)**	Variety × Light	16.59	0
**POD**	Variety	111.38	0				
	Light	424.45	0				
**(*Df* : 1;41)**	Variety × Light	115.36	0				

Table S7. Relative total antioxidant activities of phenol compounds.

Antioxidant	IC_{50} [µM]
Rosmarinic acid	2.391
Catechin	2.664
Epicatechin	3.562
Chlorogenic acid	3.660
Quercetin	3.878
Cyanidin	5.849
Ferulic acid	6.843
Caffeic acid	7.551
Eriodictyol	8.402
Ascorbate	10.328

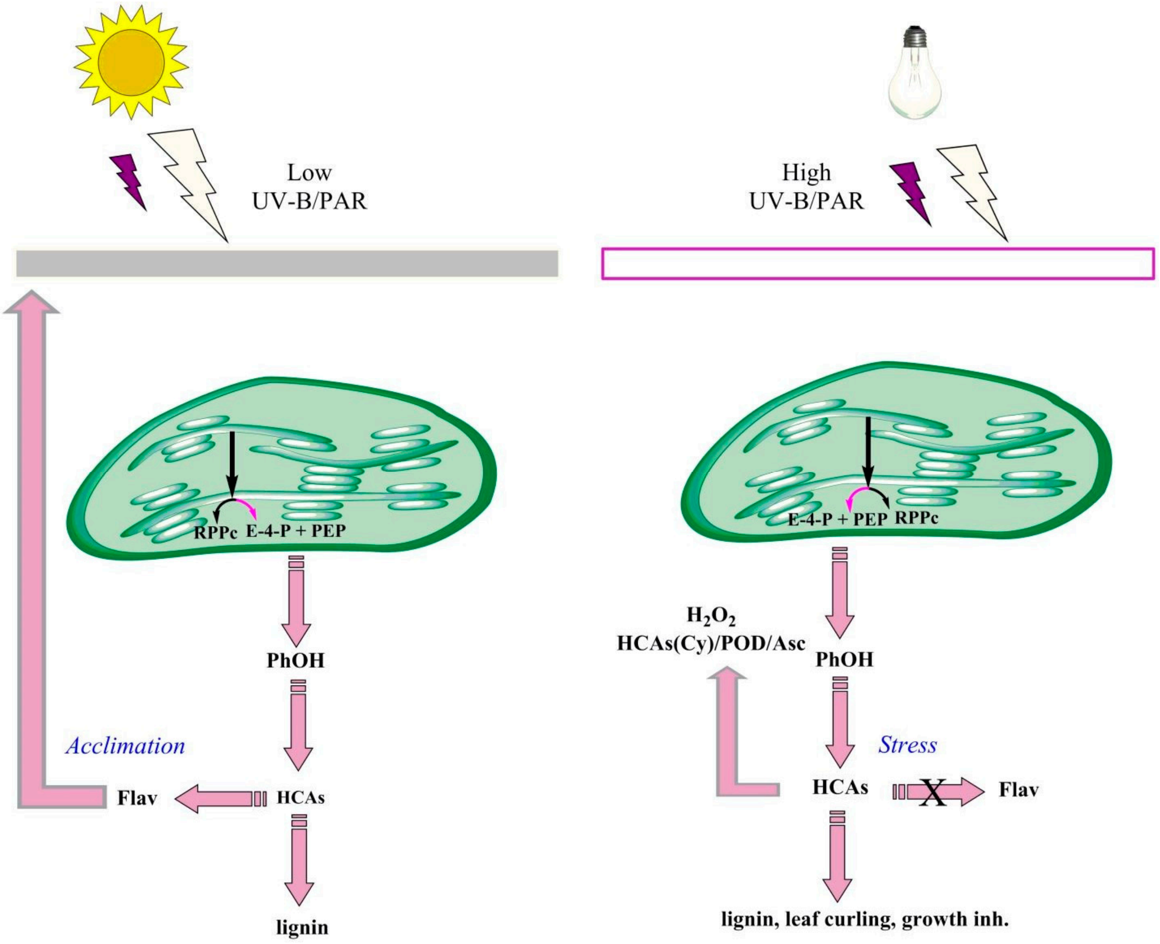

Figure S3. Schematic overview of the studied acclimative responses of three basil varieties to full sunlight from glasshouse or to UV-B compared to growth chamber. The precursors of wide range of phenylpropanoids are derived from the shikimate pathway located in chloroplasts, a key branching point between the primary and the secondary metabolism. This pathway directs carbon from glycolysis in the form of phosphoenolpyruvate (PEP) and from the reductive penthose phosphate cycle (RPP) in the form of erythrose-4-phosphate (E-4-P) towards synthesis of aromatic amino acids, phenylalanine and tyrosine, precursors for all phenolic compounds (PhOH). Both, flavonoids (Flav) and lignin are synthesized at the end of this metabolic pathway.
